# Expert design thinking workshops to analyze users’ perceived applicability of NUTRI-ONCOCARE algorithm to prevent and treat malnutrition in cancer patients under routine clinical practice conditions in Spain: the ALLIANCE study

**DOI:** 10.1007/s00520-023-08004-x

**Published:** 2023-09-01

**Authors:** Enrique Grande, Fernando Moreno, José Trigo, Jaume Capdevila, Jimena Abilés, Mariola Sirvent, Margarita Garrido-Siles, Gabriel Olveira, Julia Ocón, Maria Luisa Fernández Soto

**Affiliations:** 1 Department of Medical Oncology, MD Anderson Cancer Center Madrid, Calle de Arturo Soria, 270, 28033 Madrid, Spain; 2 Department of Medical Oncology, Hospital Clínico San Carlos, Calle del Profesor Martín Lagos S/N, 28040 Madrid, Spain; 3 Department of Medical Oncology, HC Marbella International Hospital, Ventura del Mar, 11, 29660 Marbella, Málaga, Spain; 4 Department of Medical Oncology, Vall d’Hebron University Hospital, Passeig de La Vall d’Hebron, 119, 08035 Barcelona, Spain; 5 Nutrition Unit, Hospital Costa del Sol, A-7 Km 187, 29603 Marbella, Málaga, Spain; 6 Department of Hospital Pharmacy, Clínica Vistahermosa-HLA, Avinguda de Dénia, 103, 03015 Alicante, Spain; 7 Department of Hospital Pharmacy, Hospital Universitario Virgen de La Victoria, Campus de Teatinos, S/N, 29010 Málaga, Spain; 8 Endocrinology and Nutrition Service, Instituto de Investigación Biomédica de Málaga de Avenida, Hospital Regional Universitario de Málaga and University of Malaga, Carlos Haya 84, 29010 Málaga, Spain; 9 Endocrinology and Nutrition Service, Hospital Clínico Universitario Lozano Blesa, Calle de San Juan Bosco, 15, 50009 Saragossa, Spain; 10 Department of Medicine, Hospital Universitario Clínico San Cecilio, Avenida del Conocimiento S/N, 18016 Granada, Spain

**Keywords:** Cancer nutrition, Malnutrition, ONCOCARE, NUTRI-ONCOCARE algorithm

## Abstract

**Purpose:**

NUTRI-ONCOCARE algorithm has been developed to identify and treat patients with solid tumors who are at risk of malnutrition. The present study is aimed at analyzing users’ opinion about this new tool and at assessing whether it is perceived as useful to achieve the behavioral change required for a successful integration of nutritional assessment into routine cancer care.

**Methods:**

Design thinking Double Diamond process was applied. A multidisciplinary team composed of ten potential end-users (four oncologists, three endocrinologists, one nutritionist, and two hospital pharmacists) participated in three different workshops aiming to analyze the different tasks included within the NUTRI-ONCOCARE algorithm.

**Results:**

Users agreed on the need to perform nutritional assessment around cancer diagnosis and through the course of the disease using standardized tools included in hospital nutritional protocols and involving healthcare professionals with nutrition expertise. Nutritional evaluation and intervention should be individual and comprehensive, considering not only nutritional parameters but also patients’ functional status. According to participants’ opinion, the implementation of nutritional screening in routine clinical practice is limited by the lack of time and staff to conduct nutritional assessments, the low level of nutrition expert participation, and the poor support provided by hospital managers, which are often unaware of nutrition’s impact in cancer care.

**Conclusions:**

Experts recognized the importance of considering nutritional status in cancer patients and identified the opportunity provided by the NUTRI-ONCOCARE algorithm for this purpose, as it meets main requirements for being used routinely in clinical practice.

**Supplementary Information:**

The online version contains supplementary material available at 10.1007/s00520-023-08004-x.

## Introduction

Severe *nutritional deficiencies* are detected in 15–40% of total cancer cases at the time of diagnosis, and *malnutrition* rates rise as disease progresses, affecting as much as 80% of patients at advanced stages and accounting for 20% of total cancer deaths [1–3]. Although malnutrition is a hallmark of cancer, its prevalence varies with tumor type and grade, being higher in pancreatic, gastro-esophageal, lung, and head-and-neck neoplasms, and in patients with advanced diseases [4].

In cancer patients, *undernourishment* is caused by reduced food intake, impaired nutrient assimilation, or metabolic disturbances, either directly due to the malignancy or as side effects of the oncologic treatment, both of which result in unintentional weight loss and in changes in body composition [5]. Cancer-related malnutrition can evolve into *cancer cachexia*, characterized by an ongoing loss of adipose tissue and skeletal muscle mass (known as *cancer sarcopenia*) that cannot be fully reversed by conventional nutritional support [6].

Malnutrition, cachexia, and sarcopenia adversely affect the evolution of cancer in terms of decreased patients’ quality of life (QoL) and worsened treatment outcomes [7–9]. Weight loss and reduced body mass index (BMI) are associated with shorter survival independently of the type of cancer, the patient performance status, or the disease stage [3, 10], and diminished muscle strength and function have been identified as independent predictor factors for overall survival [3, 11, 12]. In addition, malnutrition in cancer patients has been associated with a higher risk of postoperative complications [9] and with longer hospital stays, which results in significantly greater medical costs [3, 13].

Early nutritional interventions have been shown to delay the progression of malnutrition [14] and to limit its negative consequences [15]. However, studies performed across different hospitals in Europe have revealed that only 30–60% of cancer patients who are at risk of malnutrition actually receive nutritional support [4, 13, 16]. Despite the necessity to integrate nutritional care in cancer management [17], nutritional recommendations to be implemented in the clinical practice are limited [1]. Additionally, development of effective measures is conceived as complex due to the multifactorial nature of the disease and the need for personalized assessment [18].

Different quick and easy to use nutritional screening and assessment tools have been validated for both oncologic in- and outpatients [19], such as malnutrition universal screening tool (MUST) [20] and patient-generated subjective global assessment (PG-SGA) [21], but no gold standard exists. A new algorithm has been developed to evaluate the risk of malnutrition, identify malnourished patients, and establish an intervention and follow-up strategy in patients with solid tumors using NUTRISCORE, which has also demonstrated to be a simple and accurate instrument for the detection of nutritional risk in patients with cancer, with higher sensitivity and specificity rates than other available tools [22]. This algorithm is based on a prompt nutritional assessment around cancer diagnosis and a stablished follow-up of patients in order to provide nutritional intervention at early disease stages when necessary or to identify warning signs and symptoms of nutritional status worsening throughout the course of the disease [23].

This study is aimed at analyzing the applicability of NUTRI-ONCOCARE algorithm in routine clinical practice in Spain by assessing end-users’ opinion about the main tasks of the care pathway, as well as main barriers or opportunities they detect for its implementation.

## Methods

### Participants

This study was built upon the development of the NUTRI-ONCOCARE algorithm to address the prevention and treatment of malnutrition in cancer patients [23]. The NUTRI-ONCOCARE algorithm includes NUTRISCORE [22] as the nutritional screening tool, which categorizes oncologic patients based on weight loss over time, appetite in the last week, tumor location, and the treatment to be applied; considering at risk patients those with a result ≥ 5.

A multidisciplinary group composed of ten experts representing the four main clinical specialties most likely to use the algorithm (four oncologists, three endocrinologists, one nutritionist, and two hospital pharmacists) participated in the study. Enrique Grande was the coordinator of the working group for this study. Mariola Sirvent, Margarita Garrido, and Jimena Abilés, who were involved in NUTRI-ONCOCARE algorithm development, also participated as experts to provide insights into the needs, challenges, and problems regarding the use of this new tool in the clinical practice setting.

### Design

**Fig. 1:**
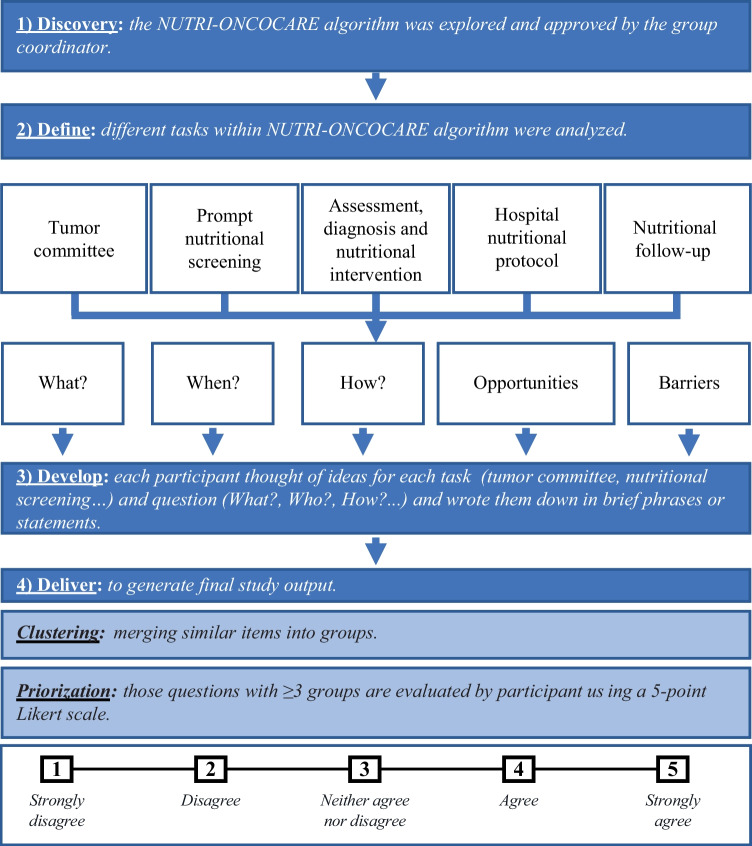
Study workflow

This study applied a user-centered *design thinking* approach, which is a problem-solving methodology that prioritizes users’ desires, needs, and challenges to develop more comprehensive and effective solutions [24]. The design thinking process followed a *Double Diamond* methodology including the following steps: *discover*, *define*, *develop*, and *deliver* (Fig. 1) [25]. First of all, as part of the *discovery* process, the NUTRI-ONCOCARE algorithm was explored by the group coordinator. The following tasks included in the NUTRI-ONCOCARE pathway were analyzed by participants: tumor committee; prompt nutritional screening; assessment, diagnosis, and nutritional intervention; hospital nutritional protocol; and nutritional follow-up. For each task, ideas were developed around the following topics: “What this task stands for?,” “When do you consider it should be applied?,” “How it should be applied?,” “Which are the main opportunities?,” and “Which are the main barriers?.” A clustering process was then performed to organize similar ideas into individual groups for each task and question. Those questions with 3 or more groups after clustering were selected for the priorization process. The relevance of each idea was evaluated by participants using a 5-point Likert scale where 1 is strongly disagree, 2 agree, 3 neutral, 4 disagree, and 5 strongly agree. Total scores were calculated to deliver final study output, which included those items rated with more than 4 in this evaluation.

### Workshop preparation and conduct

In the first meeting, held on October 19, 2021, the group coordinator (EGP) explored for the first time the adaptation of the NUTRI-ONCOCARE algorithm which had already been validated by the members of the working group who were responsible for its development (MSO, MGS, and JA). In the second meeting, which took place on 21st October 2021, the NUTRI-ONCOCARE algorithm and the methodology were presented to the working group in order to focus subsequent discussion, where each task included in the algorithm was prototyped. In the third meeting (29 November 2021), participants brainstormed ideas and wrote them down in brief statements which were arranged next to each of the tasks of the algorithm to which they referred. After the clustering process, participants were asked to evaluate the importance of each item through an online survey using a 5-point Likert scale.

## Results

Detailed information about items proposed by participants and the average scores given to each of them during the prioritization process can be found as supplementary material. Items rated with a score of more than 4 were considered relevant and are detailed below for each specific task within the NUTRI-ONCOCARE pathway.

### Tumor committee

**Fig. 2:**
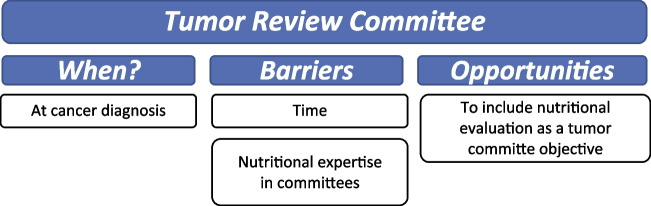
Tumor committee

Experts agreed that nutritional risk assessment should be one of the objectives of the tumor committee at the time of cancer diagnosis, thus allowing an early identification of patients who require a rapid intervention or a closer follow-up. Main barriers they identify to the implementation of these measures in routine clinical practice settle in the high workload of involved health professionals, which limits the time devoted to individual case review, as well as the lack of participation of nutrition experts in tumor committees (Fig. 2) (Supplementary table 1).

### Prompt nutritional screening

**Fig. 3:**
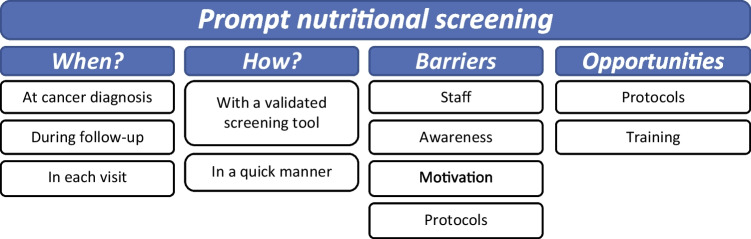
Prompt nutritional screening

Experts think that nutritional screening should be performed with a validated screening tool that takes no more than 2–3 min to be completed, in accordance with each site-specific protocols and after having received the appropriate training (Fig. 3).

According to their opinion, the unavailability of standardized protocols limits nutritional screening, as well as the lack of awareness among health-care professionals on the role of nutrition in the evolution of cancer (Supplementary table 2).

### Assessment, diagnosis, and nutritional intervention

**Fig. 4:**
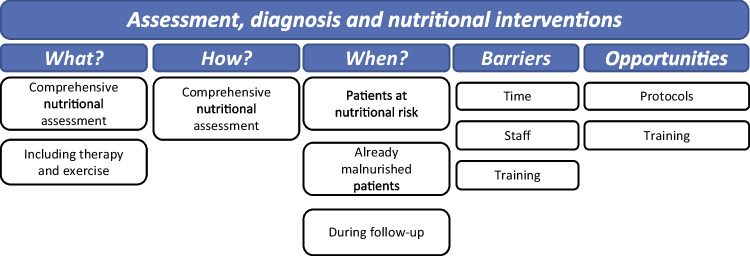
Assessment, diagnosis, and nutritional intervention

Experts support patients’ nutritional evaluation to be comprehensive, including tests for the assessment of muscle mass and function. Nutritional interventions should also consider all aspects of malnutrition and include exercise as part of the treatment strategy. Both, evaluation and intervention, are limited by the lack of time and human resources, who lack the proper training to carry it out adequately (Fig. 4) (Supplementary table 3).

### Hospital nutritional protocol

**Fig. 5:**
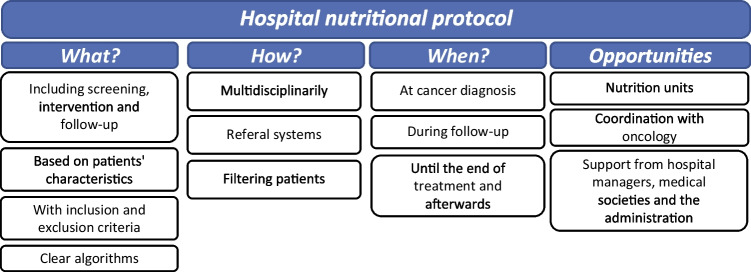
Hospital nutritional protocol

According to the opinion of experts, hospital protocols used during follow-up should include screening and intervention phases adapted to patients’ specific characteristics. Algorithms to be applied should be of ease use and clearly select patients at need of nutritional support by defined inclusion and exclusion criteria. Management of patients should be performed by a multidisciplinary group of experts with an ease referral system between clinical specialties and presence of nutrition experts. The creation of multidisciplinary nutritional units would facilitate the coordination of nutrition experts with other clinical specialties and promote support of managers, medical societies, and the administration (Fig. 5) (Supplementary table 4).

### Nutritional follow-up

**Fig. 6:**
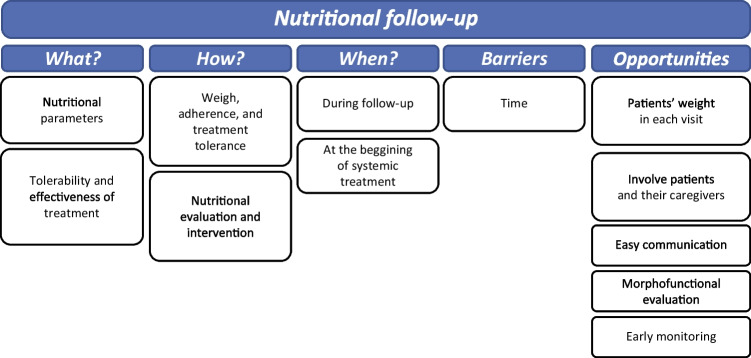
Nutritional follow-up

Experts agreed that patients should be monitored from diagnosis to the end of treatment considering nutritional parameters, as well as treatment tolerability and effectiveness. Systematic assessment throughout follow-up will allow for a continuous record of the patients’ nutritional and morphofunctional status (Fig. 6) (Supplementary table 5).

## Discussion

Despite the fact that malnutrition negatively impacts patients’ expectancy and quality of life, assessing nutritional status is not yet a common practice in cancer care. Recent efforts have focused on developing an algorithm to prevent and treat cancer-associated malnutrition. The ALLIANCE study was designed to analyze the perceived applicability of the NUTRI-ONCOCARE algorithm in routine clinical practice in Spain.

Experts recognized that nutritional evaluation should be performed around cancer diagnosis and continuously monitored through the course of the disease, what is in accordance with recommendations included in Spanish nutritional guidelines [26], which are aimed at promptly identifying patients at risk of becoming malnourished in order to perform an early treatment strategy. While malnutrition screening is a proven successful intervention, it is not an integral part of the cancer standard care pathway in most institutions, resulting in 50% of malnourished patients not being identified nor treated [19]. In a Delphi study conducted in Spain with 52 healthcare professionals experienced in cancer-related nutritional support, the assessment of malnutrition risk was reported to be performed in less than 30% of patients diagnosed with cancer. This study also revealed a high level of consensus among caregivers regarding the quality of nutrition care provided to cancer patients, which was rated as medium–low [27].

According to the opinion of experts, nutritional evaluation should be performed in the context of a multidisciplinary team, including nutrition specialists. The nutritional status of cancer patients with advanced disease has been significantly improved when dietitians, oncologists, nurses, caregivers, and patients themselves collaborate to manage the disease [28]. In this multidisciplinary context, an early involvement of dieticians resulted in a slower or stabilized weight loss in patients with different types of solid malignancies [29].

There is a need for tools that enable nutritional risk assessment to be carried out in a standardized, quick, simple, and comprehensive manner, as weight loss individually is ineffective to detect malnutrition due to its low sensitivity for metabolic changes that occur in cancer patients [30]. Although many validated screening tools are available, there is no current gold standard [31] and this has already been identified as one of the main barriers to routine nutritional assessment in clinical practice [32]. Tools such as nutritional risk screening (NRS), malnutrition universal screening tool (MUST), and patient-generated subjective global assessment (PG-SGA) have demonstrated to be efficacious for assessing unfavorable clinical outcomes in patients with cancer [33], as they consider food intake, body weight, body composition, biochemical nutritional markers, muscle function, and physical performance.

Following the NUTRI-ONCOCARE algorithm, the nutritional status of oncologic patients is evaluated around cancer diagnosis, with greater or lesser preference depending on the type of tumor. The review of each independent case by the tumor committee as the starting point of the NUTRI-ONCOCARE algorithm promotes an early nutritional assessment, and its multidisciplinary nature encourages the participation of health professionals with nutrition expertise, regardless of their medical specialty, from the beginning of the treatment pathway. Based on the assessed nutritional risk, as well as the impact of cancer therapies in the nutritional state, a specific intervention and follow-up plan is described for each patient throughout the treatment phase and beyond [23]. Nutritional risk is assessed through NUTRISCORE, in which validity in outpatients with cancer was investigated using PG-SGA as a reference method, demonstrating a sensitivity of 97.3% and a specificity of 95.9% [22].

According to criteria defined by experts, NUTRI-ONCOCARE has the right attributes to be the standard tool for hospital nutrition screenings; however, its use is not without limitations. Several of the identified barriers to NUTRI-ONCOCARE implementation in routine clinical practice have already been proposed elsewhere for general nutritional assessment in the context of cancer care. In line with our results, previous studies have revealed that oncologists are often unaware of the impact of malnutrition in the evolution of cancer and do not expect significant benefit from nutritional interventions [34–36], what has been attributed to a lack of knowledge on clinical nutrition [37]. Oncologists have reported lack of confidence in their ability to identify malnutrition and claimed additional training in this area [34]. As stated by experts participating in this study, availability of human and economic resources has also been proposed as a barrier to adequate nutrition therapy [32]. In a recent study conducted in Italy with 300 active hospital medical oncologists, shortage of time and lack of adequate personnel were identified as the main barriers to the implementation of a parallel nutritional-metabolic pathway in their institutions [38]. A low involvement of nutrition experts in cancer care is also detected, what has already been described and related to the absence of nutritional teams in hospitals [38] and to the lack of a structured collaboration between oncologists and clinical nutrition [35]. Accordingly, the NOA project, which involved eight different hospitals from the autonomous community of Andalucía, found that the non-attendance of nutrition unit staff to tumor committees (especially to those associated with a higher malnutrition risk) was one of the main barriers in the oncological nutrition process [39]. Finally, malnutrition has a notable economic impact on national healthcare systems. A study carried out in Spain revealed that hospital malnutrition is associated with substantial costs due to longer lengths of stays (11.5 days, in patients at nutritional risk at admission, versus 8.5 days in controls). The extrapolation of the potential cost of hospital malnutrition into the Spanish National Health System was of at least €1.143 billion per year [40].

Although the study includes experts from the clinical specialties which are most likely to use the algorithm, it should be noted that representativeness of the sample is limited. In line with this, a broader and more diverse audience could enhance and validate the results of this study.

In conclusion, this study reinforces the importance of nutritional screening and follow-up in oncologic patients and the opportunity provided by the NUTRI-ONCOCARE algorithm for that purpose, while highlighting the existence of certain barriers to its routine integration into cancer care pathway under real-world conditions in Spain.

## Supplementary Information

Below is the link to the electronic supplementary material.

Supplementary file1 (DOCX 33 KB)

## Data Availability

Datasets generated and analyzed during the current study are available from the corresponding author on reasonable request.
