# Supplementary material for: Expert design thinking workshops to analyze users’ perceived applicability of NUTRI-ONCOCARE algorithm to prevent and treat malnutrition in cancer patients under routine clinical practice conditions in Spain: the ALLIANCE study
Source: Support Care Cancer. 2023 Sep 1;31(9):548. doi: 10.1007/s00520-023-08004-x (PMC10474189; doi:10.1007/s00520-023-08004-x)
Supplement: Supplementary file 1 — Supplementary file1 (DOCX 33 KB) [file 520_2023_8004_MOESM1_ESM.docx]

Expert design thinking workshops to analyse users’ perceived applicability of NUTRI-ONCOCARE algorithm to prevent and treat malnutrition in cancer patients under routine clinical practice conditions in Spain: the ALLIANCE study.

Grande, Enrique^1^; Moreno Fernando^2^; Trigo, José^3^; Capdevila, Jaume^4^; Abilés, Jimena^5^; Sirvent, Mariola^6^; Garrido-Siles, Margarita^7^; Olveira, Gabriel^8;^ Ocón, Julia^9^; Fernández Soto, Maria Luisa^10^

1. Department of medical oncology, MD Anderson Cancer Center Madrid, Calle de Arturo Soria, 270, 28033 Madrid, Spain.
2. Department of medical oncology, Hospital Clínico San Carlos, Calle del Profesor Martín Lagos S/N, 28040 Madrid, Spain.
3. Department of medical oncology, HC Marbella International Hospital, Ventura del Mar, 11, 29660 Marbella, Málaga, Spain.
4. Department of medical oncology, Vall d'Hebron University Hospital, Passeig de la Vall d'Hebron, 119, 08035 Barcelona, Spain.
5. Nutrition Unit, Hospital Costa del Sol, A-7 Km 187, 29603 Marbella, Málaga, Spain.
6. Department of Hospital Pharmacy, Clínica Vistahermosa-HLA, Avinguda de Dénia, 103, 03015 Alicante, Spain
7. Department of Hospital Pharmacy. Hospital Universitario Virgen de la Victoria, Campus de Teatinos, S/N, 29010 Málaga, Spain.
8. Endocrinology and Nutrition service, Hospital Regional Universitario de Málaga and University of Malaga. Instituto de investigación biomédica De Málaga de Avenida Carlos Haya 84, 29010, Málaga, Spain.
9. Endocrinology and Nutrition Service, Hospital Clínico Universitario Lozano Blesa, Calle de San Juan Bosco, 15, 50009, Zaragoza, Spain.
10. Department of Medicine. Hospital Universitario Clínico San Cecilio, Avenida del conocimiento S/N, 18016, Granada, Spain.

**Corresponding author:** Dr. Enrique Grande, Department of medical oncology, MD Anderson Cancer Center Madrid, Calle de Arturo Soria, 270, 28033 Madrid, Spain. Telephone number: +34917878600. E-mail address: egrande@oncomadrid.com

**Supplementary table 1:** **Tumor committee**

*Each item was evaluated by participants using a 5-point Likert Scale. Selected items were those which received a score ≥4.

| **Question** | **Solution** | **Punctuation (1-5)*** |
| --- | --- | --- |
| **When?** | At diagnosis | 4.92 |
|  | Throughout the course of the disease | 3.50 |
|  | With each change in treatment | 3.17 |
|  | According to the protocol of each pathology | 3.08 |
|  | According to the protocol of each site | 2.92 |
|  | Weekly | 2.17 |
| **Opportunities** | *To introduce a specific section for the tumor committee to include nutritional risk assessment* | *4.67* |
|  | *To obtain support from hospital managers* | *4.42* |
|  | *To define criteria suggesting the need for nutritional follow-up* | *4.42* |
|  | *To select a tumor review coordinator with prior knowledge of patients* | *4.08* |
|  | *To develop an electronic information system for remote access* | *4.08* |
|  | *Time* | *4.25* |
|  | *Inclusion of nutritional evaluation as a tumor committee objective* | *4.17* |
|  | Organisation of people according to tumour type | 3.75 |
|  | Professionalisation | 3.75 |
|  | Real-time update without having to wait for the next call for proposals | 3.67 |
|  | Acknowledgement | 3.50 |
|  | Training and career incentives | 3.25 |
|  | Nurse participation | 3.00 |
| **Barriers** | *High assistance workload* | *4.67* |
|  | *Lack of time* | *4.42* |
|  | *Limited time for the tumor committee to review each individual case* | *4.42* |
|  | *Participation of nutrition experts in tumor committees* | *4.50* |
|  | *Difficulty in the presence of nutrition in all the committees* | *4.42* |
|  | Coinciding with the service's own clinical sessions | 4.00 |
|  | Lack of professionalism of the committees | 3.17 |
|  | Fluent communication | 3.00 |

**Supplementary table 2: Prompt nutritional screening**

| **Question** | **Solution** | **Punctuation**  **(1-5)*** |
| --- | --- | --- |
| Opportunities | *Site-specific protocols defining responsibilities and scope* | *4.67* |
|  | *Digitize the screening and provide training in HC* | *4.50* |
|  | *Training* | *4.17* |
|  | Create a referral protocol | 3.92 |
|  | Inclusion in the specific committee guide | 3.83 |
|  | Onco-nutrition coordination | 3.75 |
|  | Malnutrition strategy at national or autonomous community level per contract | 3.67 |
|  | Nutrition unit and oncology service. | 3.50 |
|  | Nutrition-trained staff available for screening | 3.50 |
|  | Residents should rotate between the two specialties (oncology/nutrition) | 3.00 |
| When? | *At diagnosis* | *4.50* |
|  | *At first visit and in every visit thereafter in oncology or any other clinical specialty* | *4.67* |
|  | *Throughout tumor evolution/type of tumor* | *4.17* |
|  | At the beginning or each chemotherapy cycle | 3.83 |
|  | At each hospital admission | 3.83 |
|  | Each time the patient receive treatment | 3.75 |
|  | Depending on nutritional risk | 3.75 |
| How? | *With a validated nutritional screening tool included in the malnutrition protocol of each hospital* | *4.75* |
|  | *Inclusion in patients’ medical history* | *4.75* |
|  | *Collect the result in an electronic record template* | *4.67* |
|  | *No more than 2 to 3 minutes* | *4.50* |
|  | Tool including weight loss, BMI and nutritional intake assessments | 4.00 |
|  | Minimum: weighing to measure weight loss over time and ingestion. | 3.2 |
|  | In an agile way | 3.75 |
|  | Weight, height and analytical values | 3.17 |
| Barriers | *Lack of awareness among hospital managers* | *4.50* |
|  | *Lack of staff* | *4.25* |
|  | *Motivation* | *4.42* |
|  | *Lack of a protocol* | *4.17* |
|  | *Malnutrition consequences visibility* | *4.17* |
|  | Lack of coordination between services | 3.92 |
|  | Oncologists may not know screening tools | 3.83 |
|  | Computerised system | 3.67 |
|  | Lack of practice | 3.58 |
|  | No perceived utility | 3.25 |

*Each item was evaluated by participants using a 5-point Likert Scale. Selected items were those which received a score ≥4.

**Supplementary table 3: Assessment, diagnosis and nutritional intervention**

| **Question** | **Solution** | **Punctuation**  **(1-5)*** |
| --- | --- | --- |
| What? | *Nutritional intervention, therapy, and exercise* | *4.75* |
|  | *GLIM (Global Leadership Initiative on Malnutrition) criteria use and muscle mass tests (at least dynamometry)* | *4.33* |
|  | *Comprehensive nutritional assessment* | *4.08* |
|  | Adapted to each type of tumour | 3.67 |
|  | Risk of malnutrition | 3.67 |
|  | Morphofunctional assessment | 3.67 |
| Opportunities | *Specific protocols according to the resources of each site* | *4.75* |
|  | *Algorithms easily applied to clinical practice* | *4.42* |
|  | *Number of staff* | *4.25* |
|  | *Lead nutrition experts* | *4.08* |
|  | *Easy referral systems* | *4.08* |
| When? | Patients at nutritional risk | 4.75 |
|  | Throughout the course of the disease | 4.17 |
|  | Already malnourished patients | 4.00 |
|  | In the first assessment | 3.83 |
|  | According to the specific situation of each patient | 3.75 |
| How? | *Nutritional intervention according to nutritional intake and intestinal tract functionality (EN, ONS, TPN)* | *4.50* |
|  | *Functional tests as SSPB (Summary of safety and probable benefit)* | *4.17* |
|  | *Comprehensive nutritional assessment* | *4.17* |
|  | Validated questionnaires, weight and analytical evolution | 4.00 |
|  | Muscle mass and function with dynamometry | 4.00 |
|  | Nutritional history and morphofunctional nutritional assessment | 4.00 |
|  | Hand dynamometry | 3.83 |
|  | Assessment of medical history | 3.83 |
|  | At the doctor's office | 3.75 |
|  | Integral composition assessment | 3.58 |
|  | Adapted to the patient's risk | 3.50 |
|  | Technical measures such as impedance | 3.25 |
|  | Regular visits | 3.25 |
|  | Analysis of albumin and pre-albumin, PCR | 3.17 |
|  | Classic tools and muscle echo | 2.92 |
| Barriers | Time and staff | 4.42 |
|  | Without proper screening there is no possible intervention | 4.42 |
|  | Lack of specific training in small hospitals | 4.25 |
|  | Availability of an expert for all cancer patients | 4.00 |
|  | Lack of awareness and economic provision | 3.92 |
|  | Lack of allocation of responsibilities | 3.83 |

*Each item was evaluated by participants using a 5-point Likert Scale. Selected items were those which received a score ≥4. EN: Enteral Nutrition; ONS: Oral Nutritional Supplements; TPN: Total Parenteral Nutrition.

**Supplementary table 4: Hospital nutritional protocol**

| **Question** | **Solution** | **Punctuation**  **(1-5)*** |
| --- | --- | --- |
| What? | Including screening, intervention and nutritional follow-up | 4.92 |
|  | To define the specific protocol to be used in each site according to patients’ characteristics | 4.67 |
|  | To define inclusion and exclusion criteria | 4.50 |
|  | Clear algorithms | 4.33 |
|  | Define which committees to participate in | 4.08 |
| Opportunities | Nutrition units or nutrition committees in hospitals | 4.75 |
|  | Support from hospital managers | 4.50 |
|  | Availability of committee and protocol | 4.50 |
|  | Coordination between oncology and nutrition experts mediate by a responsible | 4.17 |
|  | Strategies supported by medical societies and the administration | 4.17 |
|  | Easily accessible document in committee and consultation | 3.75 |
|  | Physical presence of nutrition consultation in the oncology area | 3.58 |
|  | Lack of referral to nutrition | 3.17 |
| When? | From the beginning, together with the screening and intervention | 4.75 |
|  | Throughtout the process | 4.50 |
|  | Until the end of treatment and afterwards | 4.33 |
|  | From diagnosis | 4.33 |
|  | When nutritional risk is present | 3.67 |
| How? | In a multidisciplinary way within the competences | 4.58 |
|  | Referral and nutritional assessment of tumours at high nutritional risk | 4.58 |
|  | Consensus with all specialists involved in the patient's treatment | 4.33 |
|  | Screening for low-risk tumours, filtering out patients | 4.25 |
|  | Presentation to the committee, discussion, approval and implementation | 4.17 |
|  | With a pre-established adaptation protocol in each hospital | 4.00 |
|  | Ease of prescribing dietary supplements | 3.58 |
| Barriers | Lack of awareness | 3.58 |
|  | Difficulty in prescribing nutritional supplements | 3.50 |
|  | Bureaucracy | 3.25 |
|  | Feeling that we are all experts without being experts | 3.25 |

*Each item was evaluated by participants using a 5-point Likert Scale. Selected items were those which received a score ≥4.

**Supplementary table 5: Nutritional follow-up**

| **Question** | **Solution** | **Punctuation**  **(1-5)*** |
| --- | --- | --- |
| What? | Weigh control, tolerability and effectiveness of prescribed treatment | 4.75 |
|  | Nutritional screening and nutritional evaluation when pathological | 4.42 |
|  | Review nutritional parameters | 4.08 |
|  | Changes in nutritional intervention objectives | 4.08 |
| Opportunities | Patient weight in each visit | 4.58 |
|  | Involve patients and their caregivers | 4.75 |
|  | Morphofunctional evaluation | 4.58 |
|  | Early monitoring | 4.58 |
|  | Easy communication between the patient and the person responsible of his/her follow-up | 4.50 |
|  | Indicated via alerts in medical records | 4.00 |
|  | Interspersing face-to-face consultation with telephone consultation | 4.00 |
|  | Providing a unique event | 4.00 |
|  | Electronic consultation with primary care | 3.92 |
|  | On-demand access to nutrition specialist or nurse | 3.83 |
| When? | Throughtout the disease | 4.92 |
|  | At the beginning of systemic treatment | 4.17 |
|  | In each oncology visit | 4.00 |
|  | In each cycle | 3.92 |
|  | Depending on oncologic treatment | 3.83 |
|  | According to baseline risk and need for support | 3.75 |
| How? | Weigh, adherence, and treatment tolerance | 4.75 |
|  | Nutritional evaluation and intervention | 4.08 |
|  | It can be established according to patient groups (surgical risk). | 3.92 |
|  | With protocol agreed by committee | 3.83 |
| Barriers | High workload | 4.67 |
|  | Lack of time | 4.33 |
|  | Lack of awareness | 3.83 |
|  | Significant improvement or worsening causes leading to loss of follow-up | 3.58 |
|  | Thinking that the nutritional monitoring is being done by another specialist | 3.25 |

*Each item was evaluated by participants using a 5-point Likert Scale. Selected items were those which received a score ≥4.
